# Localization and Functional Analysis of the *Smoothened* Gene in *Apis cerana cerana*

**DOI:** 10.3390/insects17080769

**Published:** 2026-07-27

**Authors:** Lina Guo, Yu Zhang, Chang Song, Yuan Guo

**Affiliations:** 1 College of Animal Science, Shanxi Agricultural University, Jinzhong 030801, China; linaguo@126.com (L.G.); 15183537865@163.com (Y.Z.); sconnie30@163.com (C.S.); 2 Shanxi Key Laboratory of Animal Nutrition and Feed Development, College of Animal Science, Shanxi Agricultural University, Jinzhong 030801, China; 3 College of Horticulture, Shanxi Agricultural University, Jinzhong 030801, China

**Keywords:** *Apis cerana cerana*, Smoothened, localization, function, olfactory-related genes

## Abstract

Smoothened (Smo) is a core component of the Hedgehog signaling pathway and plays an important role in insect tissue growth, but its function in honey bees remains unclear. In this study, we investigated the expression localization of *AcerSmo* in the antennae of *Apis cerana cerana (A. c. cerana)* and its impact on olfactory behavior using in situ hybridization, immunohistochemistry, RNA interference, transcriptome sequencing, and behavioral and electrophysiological techniques. The results showed that *AcerSmo* mRNA is expressed in the sensory neurons beneath trichoid and placoid sensilla, while the protein is localized to the non-neuronal supporting cells of these sensilla, revealing a spatial separation between *AcerSmo* mRNA and protein localization. Although this pattern suggests cell-type-specific post-transcriptional regulation, the present data do not directly demonstrate intercellular mRNA transport or translation. RNAi-mediated silencing of *AcerSmo* significantly upregulated the expression of *AcerOr1* and *AcerOr2*. Transcriptome sequencing identified 19 olfaction-related differentially expressed genes involved in signal transduction, substance transport, and various metabolic pathways. Electroantennogram and Y-tube olfactometer assays revealed that after *AcerSmo* interference, the responses of *A. c. cerana* to odorants changed significantly: EAG responses to 2-heptanone and hexyl acetate decreased, while avoidance behavior toward 2-heptanone increased; EAG responses to 1-nonanol and eugenol increased, and Y-tube tests showed enhanced attraction to 1-nonanol and increased avoidance of eugenol. Conclusions: These findings indicate that *AcerSmo* plays an important role in regulating olfactory receptor expression and modulating odorant responses in honey bees, thereby influencing their olfactory behavior.

## Simple Summary

The Hedgehog signaling pathway is highly conserved across species, yet its role in insect olfaction remains poorly understood. Here, we characterized the Smoothened (Smo) gene—a core component of this pathway—in the eastern honey bee *Apis cerana cerana (A. c. cerana)*. We found that *AcerSmo* exhibits a unique spatial segregation pattern: its mRNA is transcribed in sensory neurons beneath antennal trichoid and placoid sensilla, while the protein localizes to adjacent non-neuronal supporting cells, revealing a spatial separation between *AcerSmo* mRNA and protein localization, suggesting cell-type-specific regulation of *AcerSmo* expression during olfactory function. RNA interference-mediated silencing of *AcerSmo* upregulated the expression of key olfactory receptor genes *AcerOr1* and *AcerOr2*, and altered bees’ electrophysiological and behavioral responses to ecologically relevant odorants including 2-heptanone, 1-nonanol, and eugenol. Our findings demonstrate that *AcerSmo* modulates honey bee olfactory recognition by regulating olfactory receptor expression, expanding the known physiological functions of the Hedgehog pathway beyond development into adult sensory regulation.

## 1. Introduction

The Hedgehog (Hh) signaling pathway, originally discovered in *Drosophila*, is a highly conserved pathway that plays a crucial role in embryonic development and cell differentiation in both vertebrates and insects [1,2]. In *Bombyx mori*, the Hh signaling pathway participates in adult development, midgut cell proliferation, and lipogenesis [3,4]. Beyond this, *Drosophila* studies have shown that Hh signaling influences neural stem cell development, glial cell migration in the visual system, and wing development [5,6,7]. More recently, it has been established that the Hh signaling pathway regulates odorant responses by modulating the ciliary transport of odorant receptors [8,9]. Smoothened (Smo) is a core component mediating signal transduction within this pathway; however, its specific expression patterns and functions in the olfactory system of *A. c. cerana* remain largely uncharacterized. Given the established central role of Hh signaling in embryonic development and cell fate determination, we hypothesize that it may also participate in regulating key physiological processes within the adult insect olfactory system. Therefore, this study aims to investigate the Smo gene in *A. c. cerana*, specifically to verify its involvement in regulating olfactory receptor (OR) function and subsequent olfactory behavior.

The Smoothened gene (Smo) is a central component of the Hh signaling pathway and plays an essential role in its activation. In *Drosophila*, Smo significantly contributes to head formation. However, the function of the Hh signaling system, particularly Smo, in honey bees remains unclear. We previously reported high expression levels of *AcerSmo* in the antennae of *A. c. cerana*. In this study, we selected the antennae of *A. c. cerana* foragers for in situ hybridization to investigate Smo localization. We also silenced *AcerSmo* to determine its effects on *AcerOr1* and *AcerOr2* and to clarify its role in the olfactory identification process of *A. c. cerana*. The olfactory system is crucial for detecting a variety of volatile chemicals in the environment, including compounds produced by prey, host plants, and conspecifics [10]. Each olfactory neuron typically expresses two types of odorant receptors: conventional odorant receptors (ORs) and a highly conserved olfactory receptor coreceptor (Orco). ORs are essential for olfactory recognition in insects [11]. In insects, odorant receptors (ORs) function together with the highly conserved odorant receptor co-receptor (Orco) to form heteromeric ligand-gated ion channels located on the dendritic membranes of olfactory sensory neurons. Upon odorant binding, activation of the OR-Orco complex initiates neuronal depolarization and downstream olfactory signal transduction [12,13,14]. *A. c. cerana*, a species of bee native to China, plays significant roles in both economic and ecological contexts [15]. *A. c. cerana* has a greater number of antennal olfactory receptors and expresses its odor receptor coreceptor (Orco) at higher levels than Apis mellifera Linnaeus, indicating heightened sensitivity to volatile floral chemicals [16]. *AcerOr1*, a traditional odorant receptor gene, and *AcerOr2*, its coreceptor (Orco) gene, substantially contribute to the olfaction of *A. c. cerana* [17,18].

RNA interference (RNAi), a highly conserved post-transcriptional gene-silencing mechanism, has become one of the most effective reverse-genetic tools for functional gene characterization in insects [19]. By enabling sequence-specific suppression of target gene expression, RNAi has been widely used to investigate the physiological and behavioral functions of gustatory receptors, olfactory receptors, and other sensory genes in diverse insect species. More recently, the application of RNAi has extended far beyond functional genomics. Owing to their high target specificity, environmental compatibility, and reduced non-target effects, RNAi-based pesticides have emerged as a promising next-generation pest management strategy and have been regarded as the “third revolution” in pesticide development following chemical and biological pesticides [20,21]. In apiculture, RNAi technology has also shown considerable potential for controlling honey bee pathogens and parasites, particularly Varroa destructor and several viral diseases, with dsRNA-based products already entering commercial development [22]. Therefore, functional identification of honey bee gustatory receptor genes not only advances our understanding of the molecular mechanisms underlying taste perception but also provides valuable molecular targets for future RNAi-based applications in pollinator health management.

In this study, candidate gustatory receptors were screened by molecular docking and validated by RNAi, PER and behavioral assays to elucidate the molecular mechanisms of sweet and bitter perception in *A. c. cerana*. This study provides functional evidence for honeybee gustatory receptors and lays a foundation for future RNAi-based applications.

## 2. Materials and Methods

### 2.1. Experimental Insects

*A. c. cerana* used in the experiment were obtained from the apiary of the College of Animal Science of Shanxi Agricultural University (Taigu, China). Between 09:00 and 11:00, bees carrying pollen on their hind legs were randomly captured at the entrance to the *A. c. cerana* hives. Using sterilized tweezers, we gently immobilized these pollen-collecting bees on their thoraxes. We then placed them into wooden boxes and incubated them at a constant temperature and humidity for subsequent experiments.

### 2.2. In Situ Hybridization

After capturing live foragers, their thoraxes were gently held with forceps, and their antennae were carefully excised using a sharp scalpel. The antennae were then promptly submerged in a fixative solution containing 2.5% glutaraldehyde for 2 h. Following fixation, they were dehydrated through a graded ethanol series (25%, 50%, 75%, 95%, and 100%), with 20 min of incubation at each concentration. Once fully dehydrated, the antennae were embedded in paraffin and sectioned at a thickness of 5 μm. The resulting sections were mounted on slides, dried at 62 °C for 2 h, and deparaffinized with sequential Xylene treatments (15 min in Xylene I, followed by 15 min in Xylene II. The sections were then rehydrated by immersion in anhydrous ethanol I for 5 min, anhydrous ethanol II for 5 min, 85% ethanol for 5 min, and 75% ethanol for 5 min, with rinsing in diethyl pyrocarbonate (DEPC)-treated water after each step.

Next, the sections were immersed in antigen retrieval solution (Sangon Biotech, Shanghai, China)—a sodium citrate buffer (citric acid and sodium citrate, pH 6.0)-which was heated to boiling for 10–15 min. After boiling, the sections were rinsed with DEPC-treated water and allowed to cool naturally. A Pap Pen (Sangon Biotech, Shanghai, China) was then used to circumscribe areas of interest on the sections. Proteinase K (20 μg/mL) was added dropwise to digest the sections for 30 min at 37 °C. Following digestion, the sections were washed thoroughly with clean water and then rinsed three times in phosphate-buffered saline (PBS) for 5 min each. A prehybridization solution-comprising 50% deionized formamide, 5% 50× Denhardt’s solution, 5% tRNA (20 mg/mL), 15% DEPC-treated water, 10% dextran sulfate, and 15% buffer salt solution (3 M NaCl, 100 mM Tris–HCl, pH 8.0, 50 mM EDTA, 100 mM sodium phosphate)—was added dropwise and incubated for 1 h at 37 °C before removal. Hybridization solution (prehybridization solution supplemented with the probe at 8 ng/μL) was then applied, and the sections were hybridized overnight at 37 °C in a thermostat oven. Post-hybridization, the sections were washed as follows: Incubate the sample in 2× SSC at 37 °C for 10 min, perform two successive 5 min washes in 1× SSC at 37 °C, and then incubate in 0.5× SSC at room temperature (21–30 °C) for 10 min. Subsequently, add DAPI counterstain (2 μg/mL in PBS) dropwise and stain in the dark for 8 min, followed by mounting with anti-fade mounting medium. Finally, the sections were observed and imaged using an orthogonal fluorescence microscope (Nikon Instruments Inc., Tokyo, Japan) with 4×, 10×, and 40× objectives and a10× eyepiece. Imaging wavelengths were set to 330–380 nm excitation/420 nm emission for blue (UV/DAPI) and 510–560 nm excitation/590 nm emission for red (CY3). The probe, labeled with a fluorescent FITC group at its 5′ end (sequence:5′-FITC-CCAGCAAAGAAAGGUGAUAGUAAAUGUAAC-3′), was synthesized by Sangon Biotech (Shanghai, China).

### 2.3. Immunohistochemistry

The antennal tissues were embedded in OCT compound and sectioned using a cryostat. The sections were air-dried at room temperature, then baked in an oven at 37 °C for 10–20 min. Slides were incubated at 37 °C only to facilitate tissue drying before subsequent staining rather than for antigen retrieval or tissue fixation. Subsequently, the sections were fixed in methanol for 20 min and washed three times with PBS (pH 7.4), each for 5 min, on a decolorizing shaker. The slides were then incubated in a 3% hydrogen peroxide solution for 25 min at room temperature in the dark to block endogenous peroxidase activity, following a protocol routinely applied in insect immunohistochemistry [23]. Following incubation, the slides were washed three times with PBS (pH 7.4), for 5 min each on a decolorizing shaker. After draining, the sections were encircled with an immunohistochemistry pen. A 3% BSA solution was applied evenly within the circle to cover the tissue, and the sections were blocked for 30 min at room temperature. The blocking solution was then discarded, and the sections were incubated with a primary antibody (rabbit anti-AcerSmo polyclonal antibody, custom-made, produced by ZoonBio Biotechnology (Nanjing, JiangSu, China), catalog number ZD2022N018133-3) diluted 1:100 in PBS. The sections were placed flat in a humidified chamber and incubated overnight at 4 °C. The slides were then washed three times for 5 min each on a shaker in PBS (pH 7.4). After slight draining, an HRP-labeled secondary antibody (goat anti-rabbit) corresponding to the primary antibody was applied within the circle to cover the tissue, and the sections were incubated for 50 min at room temperature. The slides were washed three times for 5 min each on a shaker in PBS (pH 7.4).

After slight draining, freshly prepared DAB chromogenic solution (Servicebio, Wuhan, China) was applied within the circle. Color development was monitored under a microscope, with a positive result indicated by a brownish-yellow color. Once the desired coloration was achieved, the reaction was terminated by rinsing the sections with tap water. The sections were counterstained with hematoxylin for approximately 3 min, rinsed with tap water, differentiated in hematoxylin differentiation solution for a few seconds, rinsed again with tap water, treated with hematoxylin bluing solution, and finally washed under running water.

The sections were then dehydrated and cleared by sequential immersion in the following solutions: 75% alcohol for 5 min, 85% alcohol for 5 min, absolute ethanol I for 5 min, absolute ethanol II for 5 min, n-butanol for 5 min, and xylene I for 5 min (n-Butanol dehydration was included because it improves paraffin infiltration of insect tissues containing abundant cuticular structures and enhances tissue preservation). The slides were removed from xylene, allowed to dry briefly, and mounted with mounting medium. The mounted sections were observed under an optical microscope (Nikon Instruments Inc., Tokyo, Japan) to interpret the result.

### 2.4. Synthesis and Feeding of dsRNA

Based on the *AcerSmo* gene sequence (OP616035), we designed and synthesized two distinct interference fragments, dsAcerSmo-1 and dsAcerSmo-2, along with a non-targeting dsRNA fragment (NC), whose sequence shows no significant homology to any known *A. c. cerana* gene according to BLAST (Blast.ncbi.nlm.nih.gov, accessed on 22 July 2026) analysis. Forager bees were gently clamped at the thorax with sterilized forceps and placed into eight wooden boxes (12 cm × 12 cm × 5 cm), with 25 bees per box. The eight boxes were divided into four treatment groups, with two boxes in each group. After starving the bees for 0.5 h, they were fed 30% sugar water containing the interference fragments dsAcerSmo-1, dsAcerSmo-2, or the NC fragment, dissolved in the sugar water. A blank control group (CK) was included, in which bees received only 30% sugar water. Based on our previous studies, after grasping and starving the bees for 0.5 h, each bee was individually fed 20 μL of 30% sucrose solution containing 8 μg dsRNA (0.4 μg/μL). Bees that failed to consume the entire solution were excluded from subsequent analyses. Each individual bee was regarded as one biological replicate for all statistical analyses. Bee mortality was monitored daily throughout the experimental period, and no abnormal mortality was observed. After consuming the sugar water containing the dsAcerSmo or NC fragments, the bees were given an additional 30% sugar water without dsRNA or NC fragments until the end of the experiment. The boxes were then placed in a temperature- and humidity-controlled incubator set to 28 ± 0.5 °C and 65% ± 5% relative humidity, for incubation periods of 1, 2, 3, 4, and 5 days. On each day, eight bees were collected separately from each treatment group. The collected bees were rapidly frozen in liquid nitrogen and stored at −80 °C for future use.

### 2.5. Quantitative Reverse-Transcription PCR

The silencing effect was evaluated based on the expression level of *AcerSmo* in each group. *AcerOr1* and *AcerOr2* were selected to evaluate the effect of *AcerSmo* silencing based on our previous work and their distinct, complementary roles in the *A*. *c*. *cerana* olfactory system. Specifically, *AcerOr2* encodes the highly conserved olfactory co-receptor (Orco), which is essential for the formation of functional odorant receptor (OR) complexes, while *AcerOr1* acts as a specific odorant-tuning receptor implicated in floral odor detection. Together, they represent the two essential components required for odor detection. Therefore, monitoring their expression levels provides a reliable and representative readout for assessing how *AcerSmo* silencing impacts downstream olfactory pathways. The expression of the expression levels of selected olfactory receptor genes were analyzed following RNAi treatment. *AcerOr1* and *AcerOr2* in *A. c. cerana* were assessed using gene sequences (*Acer*Arp1:HM640276.1, *AcerOr1*:JN792580, *AcerOr2*:JN792581) retrieved from NCBI (Table 1). Total RNA was extracted using TRIzol^TM^ reagent (Thermo Fisher Scientific, Waltham, MA, USA) following the manufacturer’s instructions. First-strand cDNA was synthesized from 1 μg of total RNA using the PrimeScript^TM^ RT Reagent Kit with gDNA Eraser (Perfect Real Time) (Takara Bio, Kusatsu, Japan). Quantitative reverse transcription PCR (RT-qPCR) was performed using the TB Green^®^ Premix Ex Taq^TM^ (Tli RNaseH Plus) kit (Takara Bio, Beijing, China) and the CFX Connect Real-Time PCR Detection System (Bio-Rad Laboratories, Hercules, CA, USA). Each sample included three technical replicates and three biological replicates. The 10 μL reaction mixture contained 5 μL of TB Green Premix Ex Taq II (Tli RNaseH Plus) (Takara), 1.4 μL of cDNA, 0.3 μL of each forward and reverse primer, and 0.3 μL of double-distilled water (ddH_2_O). The reaction protocol consisted of 40 cycles of 95 °C for 3 min, 58 °C for 30 s, and 72 °C for 30 s. The melting curve was determined with increments of 0.5 °C from 52 to 95 °C. The comparative 2^−ΔΔCt^ method was used to analyze the results. One-way analysis of variance (ANOVA) followed by Duncan’s multiple range test (SPSS 25.0; IBM, Armonk, NY, USA) was employed to determine the statistical significance of differences, with significance set at *p* < 0.05. In this study, Arp1 from *A. c. cerana* (GenBank accession number: NM_001185145.1) was used as the reference gene.

### 2.6. Transcriptome Sequencing

Based on the results of RT-qPCR, we selected the interfering fragment ds*r*AcerSmo-1 and fed the bees for 1 day. Groups of 10 bees were fed with 30% sugar water containing dsAcerSmo-1 (RNAi), a negative control (NC) fragment, or sugar water only (CK) for 1 day. Each treatment group (CK, NC and RNAi) consisted of three independent biological replicates, with each replicate comprising pooled antennae collected from individual bees. Total RNA was extracted from the heads (including antennae) of worker bees using TRIzol reagent (Invitrogen, Carlsbad, CA, USA) following the manufacturer’s protocol. Three RNA samples per group, designated as CK1-3, NC1-3, and RNAi1-3, were pooled from five worker bees each. RNA quality and integrity were assessed using 1% agarose gel electrophoresis, a NanoDrop 2000 spectrophotometer (Thermo Fisher Scientific, Waltham, MA, USA), and the RNA Nano 6000 Assay Kit (Agilent Technologies, Santa Clara, CA, USA) on an Agilent Bioanalyzer 2100 (Agilent Technologies). mRNA was enriched, fragmented, and reverse transcribed to synthesize cDNA. Libraries were constructed with Illumina adapters, enriched, and sequenced on the Illumina HiSeq^TM^ platform, generating 100-base-pair paired-end reads. Library construction, sequencing, and raw data quality control were performed by Sangon Biotech (Shanghai, China) Co., Ltd. The Apis cerana reference genome and its corresponding genomic annotation file (GCF_001442555.1_ACSNU-2.0) were downloaded from the NCBI FTP server (https://ftp.ncbi.nlm.nih.gov/genomes/all/GCF/001/442/555/GCF_001442555.1_ACSNU-2.0, accessed on 20 July 2026) and used as the reference sequences for alignment. Quality-controlled reads were aligned to the reference genome using HISAT2v2.2.1, and the resulting alignments were evaluated using RSeQC v5.0.4. Transcripts were assembled using StringTie software v2.1.6, and gene expression levels were quantified as TPM (Transcripts Per Million). Subsequently, Differential gene expression among the comparison groups was analyzed using DEGseq v1.30.1. For differential expression analysis, the screening criteria were set as follows: a false discovery rate (FDR) ≤ 0.05 and an absolute log2 (Fold Change) (FC) ≥ 1.2. An FC value > 0 indicated up-regulated expression, while an FC value < 0 indicated down-regulated expression.

To understand the functional roles of differentially expressed genes (DEGs) that were significantly upregulated or downregulated before and after RNA interference (RNAi), their annotations from the Gene Ontology (GO) and Kyoto Encyclopedia of Genes and Genomes (KEGG) databases were utilized. Functional enrichment analysis was performed using the top GO and clusterProfiler packages. A functional term or pathway was considered significantly enriched if its adjusted *p*-value (Q-value) was <0.05.

### 2.7. Electroantennography Response to Compounds After RNAi Silencing

For electroantennogram (EAG) measurement in *A. c. cerana*, we selected odorants that previously elicited sensitive responses, based on our earlier findings [21] (Table 2). To investigate the role of specific genes in olfaction, *AcerSmo* was selected as a target gene for silencing. RNA interference (RNAi) was performed, and the silencing efficiency was subsequently confirmed by RT-qPCR. For the EAG experiments, solutions of various odor chemicals were prepared by dissolving them in paraffin to a concentration of 500 µg/µL. A 10 μL aliquot of the 500 μg/μL odorant stock solution was loaded onto each filter paper strip and inserted into the stimulus cartridge. Subsequently, antennae were extracted and their sensitivity to volatiles was assessed using an electroantennogram setup (Ockenfels Syntech GMBH, Buhenbach, Germany).

After the signal had stabilized and before testing, the antennae were carefully excised using a scalpel. An appropriate amount of SpectraR360 electrode adhesive was applied to secure the electrodes to the antennae: the reference electrode was attached to the base, and the recording electrode was connected to the tip. This setup produced a relatively Smooth baseline on the recording trace. A 20 μL aliquot of the odorant solution in liquid paraffin was applied to a filter paper strip (3 cm × 1.5 cm) and inserted into a Pasteur pipette for stimulus delivery. The control stimulus was presented once before and once after each sample test. Each test compound underwent three technical replicates on the same antenna. The entire experiment was repeated at least six times to ensure reliability.

The responses of bees to volatiles were quantified using relative electroantennogram (rEAG) values, These values are expressed as means ± standard error and calculated according to the following formula: rEAG (%) = (EAG(X) − EAG(std))/EAG(std), where EAG(X) represents the amplitude (in millivolts, mV) of the EAG response to a test compound, and EAG (std) represents the amplitude (in millivolts, mV) of the EAG response to the corresponding reference liquid paraffin from each recording session.

### 2.8. Behavior Tests

Test chemicals for the olfactory behavior assays were selected based on their strong electroantennogram (EAG) responses in the *A. c. cerana* antennae (Table 2). The Y-tube olfactometer consisted of one main arm (20 cm) and two side arms (20 cm each), with an internal diameter of 2.5 cm and an angle of 60° between the two side arms. The concentration of the tested substances matched that used in the EAG recording. Following *AcerSmo* silencing, bees were transferred to a dark room maintained at 28 ± 2 °C for behavioral assays. All tests were conducted between 08:00 and 12:00 in a dark room equipped with a Y-tube olfactometer (Nanjing XueLai Biotechnology Co., Ltd., Nanjing, China). Before each experimental session, the gas pump was activated for 1 min to establish airflow through the olfactometer. Bees were introduced individually at the center junction of the Y-tube olfactometer to assess their odor selectivity. Each bee was observed for a minimum of 5 min. A choice was recorded when a bee either entered an odor source bottle or remained within the final one-third of an arm for at least 4 min. After testing every five bees, the odor source bottles, including both odorant samples and pure liquid paraffin, were replaced. This practice aimed to maintain the integrity of the odor cues and prevent desiccation of the odorants. The experiment comprised a total of five sessions, with 10 bees tested in each session. To mitigate potential positional bias and ensure the robustness of the results, the olfactometer was either inverted or replaced after every two bees. Furthermore, after each odorant sample test (i.e., each specific odorant vs. control comparison), both the odor source bottle and the entire olfactometer were thoroughly washed with 75% alcohol followed by distilled water, and then dried completely. This rigorous cleaning protocol was implemented to eliminate any residual scents and prevent carryover effects between subsequent trials.

The convergent selection rate was calculated using the following formula: Convergent Selection Rate (%) = (Number of bees choosing a specific odor/Total number of bees tested) × 100%.

### 2.9. Statistical Analysis

Relative gene expression levels were calculated using the 2^−ΔΔCt^ method, where F = 2^−ΔΔCt^. The ΔCt values were determined as follows: ΔCt(treatment group) = (Mean Ct of target gene in treatment group − Mean Ct of reference gene), ΔCt (control group) = (Mean Ct of target gene in control group − Mean Ct of reference gene). Subsequently, ΔΔCt was calculated as ΔCt (treatment group) − ΔCt (control group). Two-way ANOVA, followed by Duncan’s multiple range test, was performed using SPSS 27.0 software (IBM Corp., Armonk, NY, USA) to determine significant differences, with *p* < 0.05 indicating statistical significance. All figures were generated with GraphPad Prism 8.0.2 and assembled using Visio 2024. The relative EAG response (rEAG), attraction rate, and repellent rate were calculated according to Equation (1). Significant differences in EAG responses were determined by one-way ANOVA followed by post hoc Duncan’s test. Behavioral data were analyzed using the chi-square(χ^2^) test in SPSS25.0 software, with significance set at *p* < 0.05.

(1) $${RV}_{EAG}=\frac{V_{S}-V_{b}}{V_{b}}$$

NOTE: *V_S_* is the EAG response amplitude induced by the test sample, and *V_b_* represents the average response amplitude of the two successive solvent controls (before and after odor stimulation).

## 3. Results

### 3.1. mRNA Localization of AcerSmo via In Situ Hybridization

**Figure 1:**
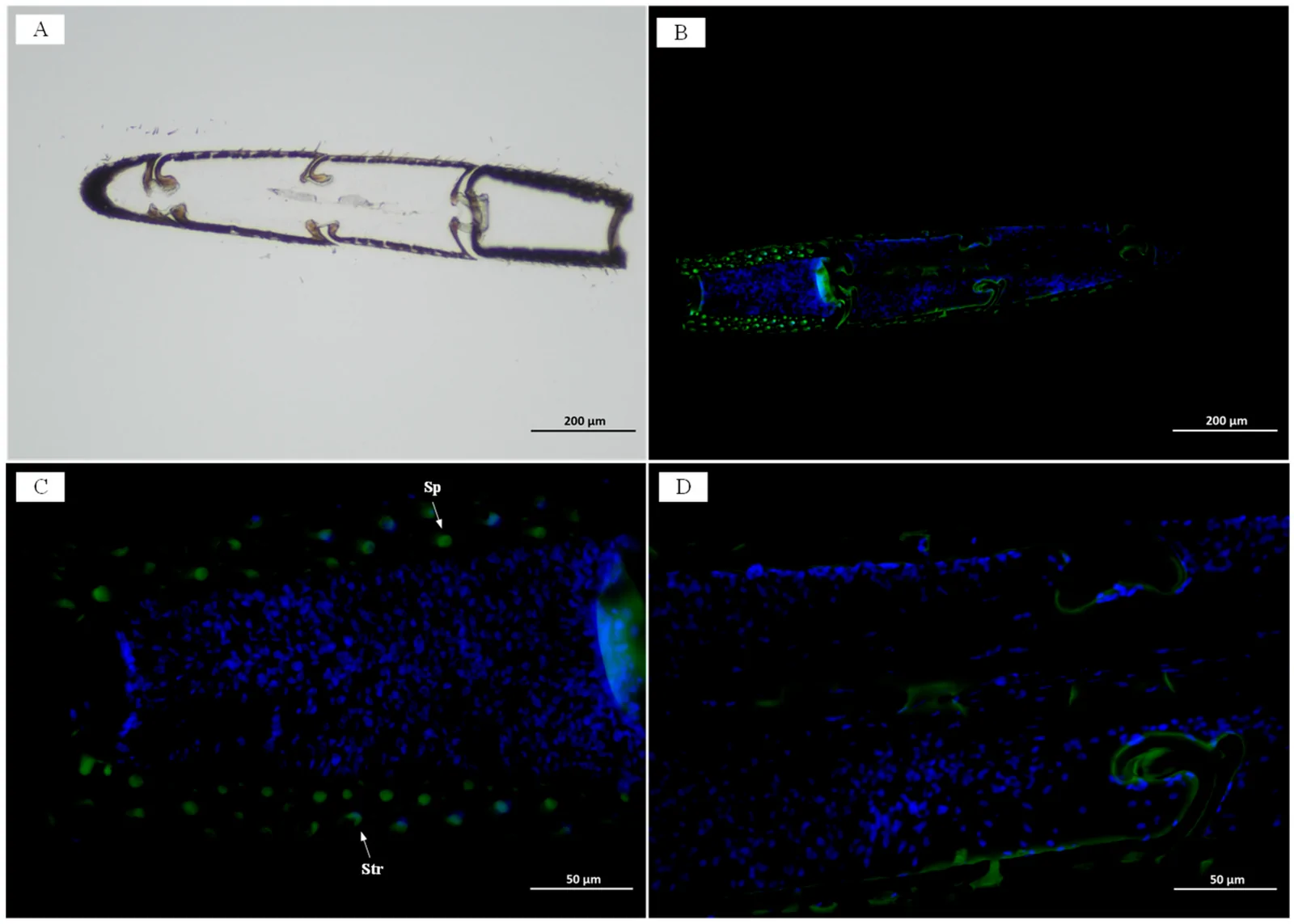
Localization of *AcerSmo* in the antennae of *A. c. cerana.* Note: (**A**) Unhybridized antenna of *A. c. cerana* (negative control); (**B**) Fluorescence localization of *AcerSmo* under an upright microscope at 200× magnification. Green fluorescence indicates the specific distribution of *AcerSmo* in the antenna of *A. c. cerana*, while blue fluorescence marks DAPI-stained nuclei; (**C**,**D**) higher-magnification views (400×) of the target region in (**B**) under an upright microscope. Str represents the sensilla trichodea; Sp represents sensilla placodea.

AcerSmo hybridization signals were widely distributed across each subsegment of the antennal flagellum of *A. c. cerana* (Figure 1B). The green fluorescence signals of the *AcerSmo* gene highly overlapped with the blue fluorescent signals of DAPI-stained nuclei and were not distributed in the epidermal cuticle lacking nuclear signals, suggesting that *AcerSmo* expression is localized within cells rather than in the epidermal cuticular structures. In Figure 1C, the overall green fluorescence pattern highly matched the distribution characteristics of the sensilla trichodea and sensilla placodea on the antennal epidermis, with signals specifically enriched in the neuronal layer beneath the base of both sensillum types, suggesting that *AcerSmo* is associated with olfactory sensory structures. AcerSmoFurthermore, *AcerSmo* exhibited significantly high expression in the subsegments near the base of the antennal flagellum, suggesting its potential involvement in the regulation of multiple sensory perception processes mediated by the antenna.

### 3.2. Identification and Titer Determination of Polyclonal Antibody

**Figure 2:**
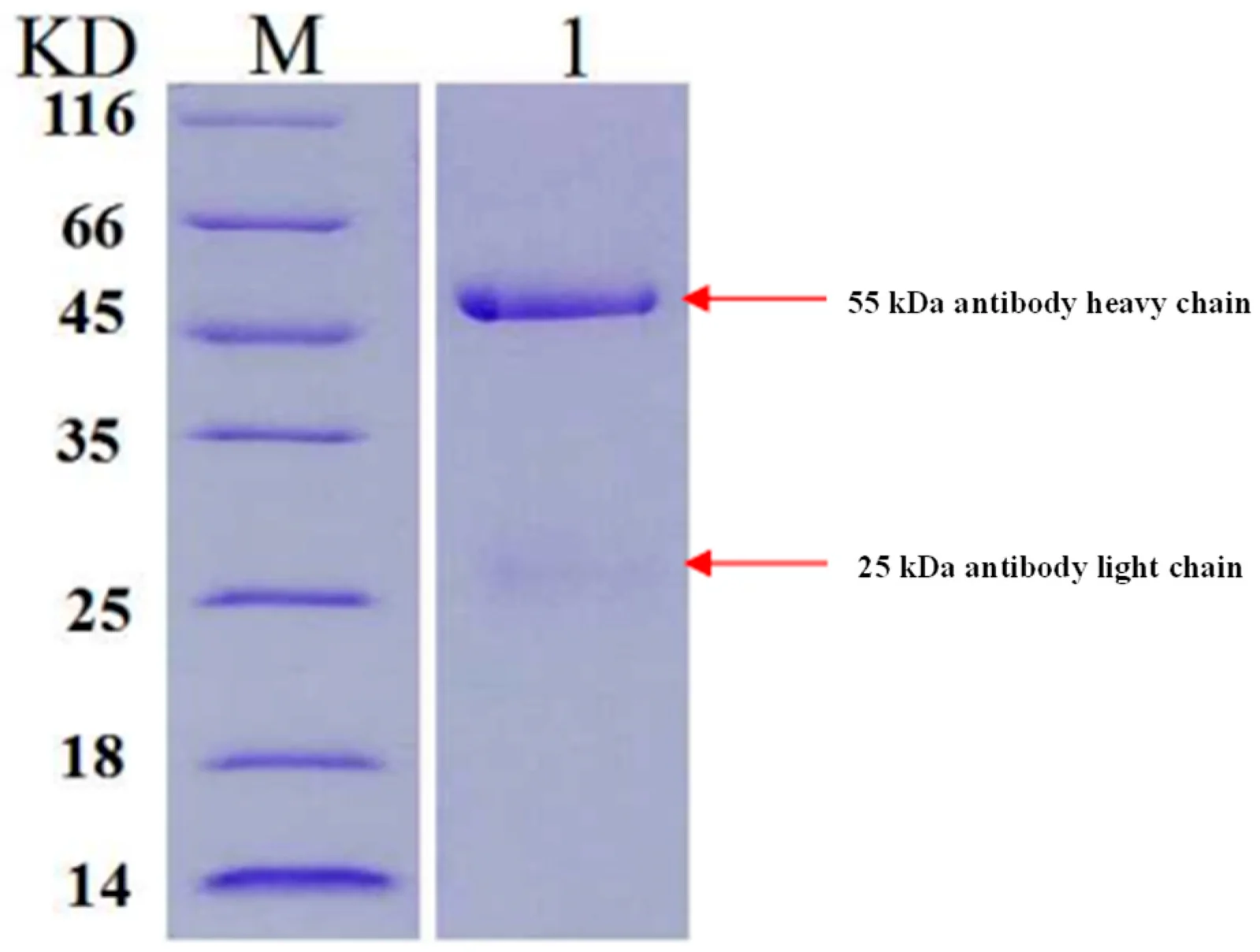
SDS-PAGE analysis of *AcerSmo* antibodies. Note: Lane M: protein marker, lane 1: *AcerSmo* antibody.

SDS-PAGE electrophoresis of the purified antibody, followed by Coomassie Brilliant Blue staining, revealed a purity exceeding 85% (Figure 2). ELISA analysis determined that the *AcerSmo* antibody titer was greater than 364,500, and the total yield of the purified antibody reached over 26.4 mg (Table 3). These results collectively indicate that the antibody was suitable for subsequent experimental applications.

### 3.3. Spatial Distribution of AcerSmo Protein Detected by Immunohistochemistry

**Figure 3:**
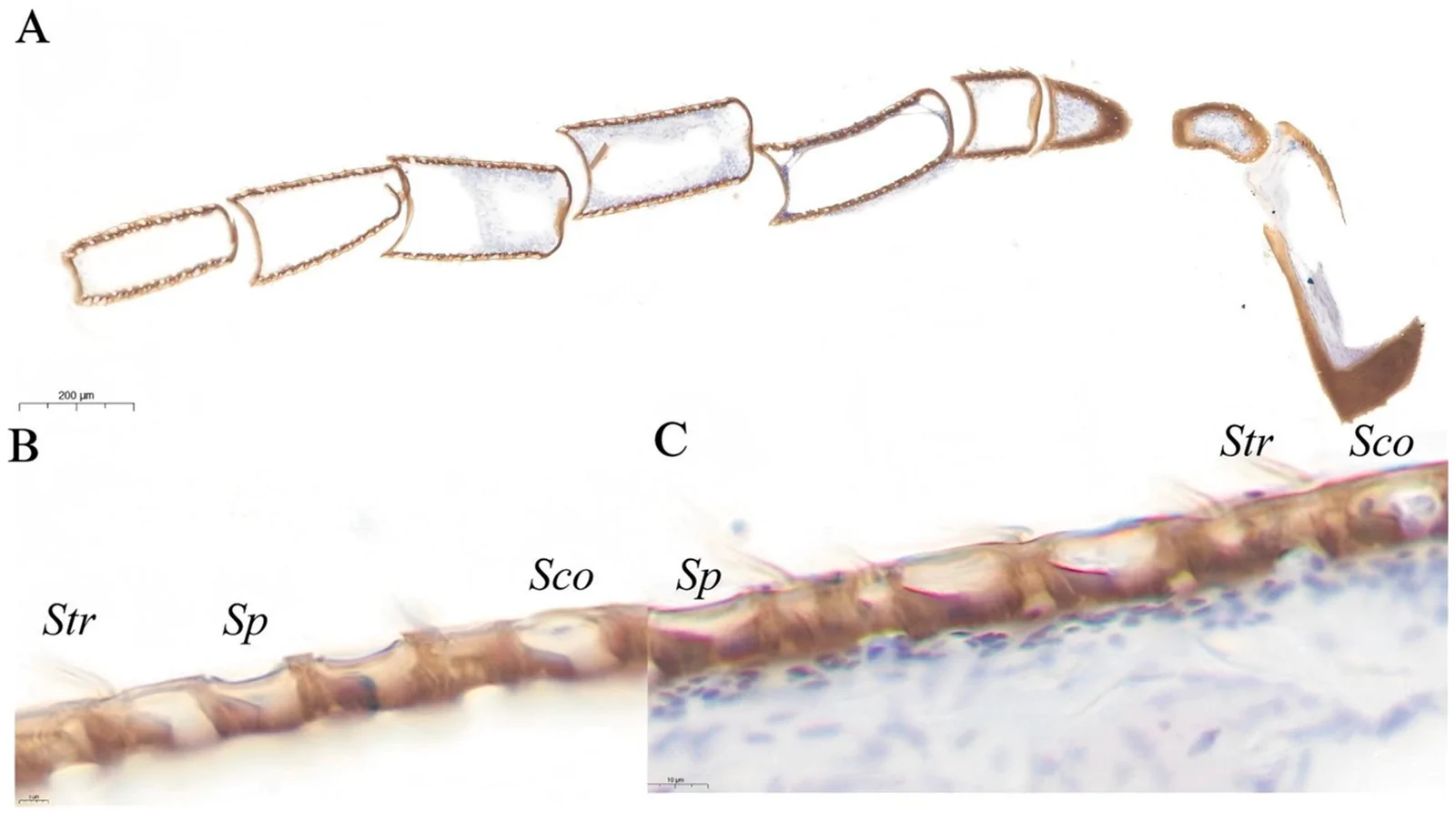
The expression of protein *AcerSmo* in *A. c. cerana*. Note: (**A**) represents a complete longitudinal section of the antenna of *A. c. cerana*; (**B**) represents a partial magnification of a flagellar subsegment; (**C**) represents a high-magnification view of the antennal cuticle. Str: Sensilla trichodea; Sp: Sensilla placoid; Sco: Sensilla coeloconica. Hematoxylin stains cell nuclei blue, and the positive expression detected by DAB appears brownish-yellow.

Immunohistochemical analysis revealed that *AcerSmo* protein was expressed in longitudinal sections of the scape, pedicel, and flagellum of *A. c. cerana* (Figure 3A). Its distribution was observed in the subepidermal tissues adjacent to the cuticle. Notably, higher protein levels were detected in the proximal subsegments of the flagellum, which may be correlated with the abundant sensilla distribution in these regions. In contrast, the apical terminal subsegments of the flagellum exhibited only extremely weak *AcerSmo* immunoreactivity. Further evaluation of *AcerSmo* protein localization within the antennal sensilla revealed strong immunoreactivity in cells adjacent to trichoid, placoid, and coeloconic sensilla (Figure 3B). Among these, *AcerSmo* was expressed within the coeloconic sensilla, whereas in the trichoid and placoid sensilla, it was localized to non-neuronal supporting cells, which are likely accessory cells associated with olfactory sensory neurons.

### 3.4. Detection of AcerSmo Silencing Efficiency

**Figure 4:**
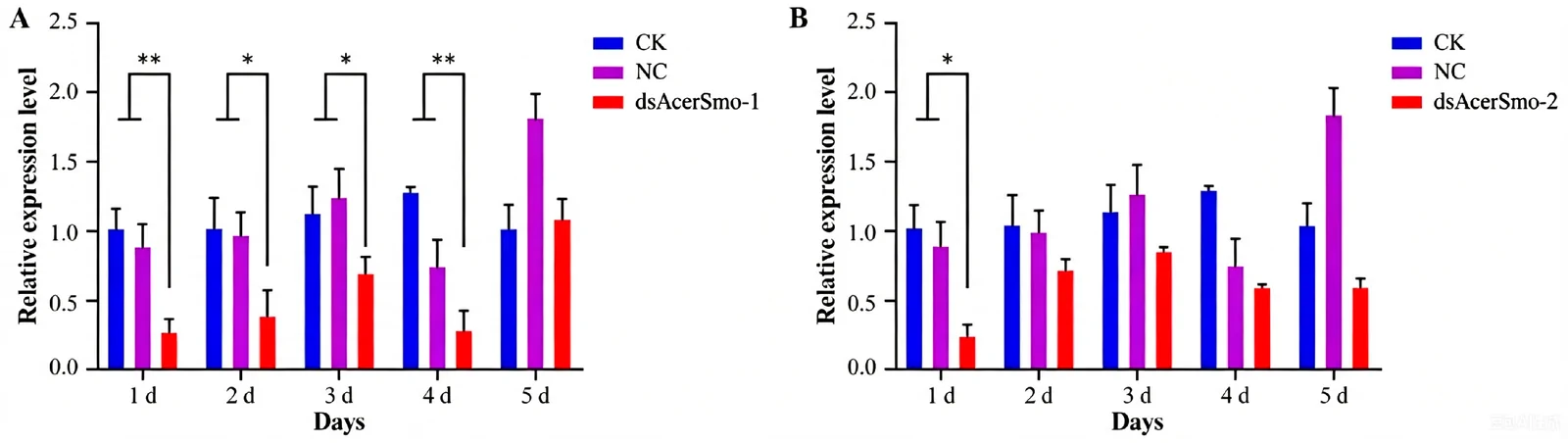
Expression analysis of *AcerSmo* transcript patterns following various treatments. CK: blank control group; NC: negative control dsRNA group; ds*AcerSmo*-1:*AcerSmo* dsRNA interference group. (**A**) dsAcerSmo-1 treatment group; (**B**) dsAcerSmo-2 treatment group. * *p* < 0.05, ** *p* < 0.01.

To evaluate the silencing efficiency of two independent dsRNA fragments, qPCR was utilized to quantify the expression levels of *AcerSmo* mRNA in bees fed with double-stranded RNA (dsRNA) fragments, dsAcerSmo-1, dsAcerSmo-2, a negative control dsRNA fragment (NC), and sugar water (CK) over a 1 to 5 day period. The results indicated that both dsAcerSmo-1 and dsAcerSmo-2 fragments significantly reduced the *AcerSmo* mRNA expression to varying degrees (Figure 4). Specifically, from day 1 to day 4 post-treatment (*p* < 0.05), *AcerSmo* expression in the AcerSmodsAcerSmo-1-fed group was significantly lower than that in the sugar water (CK) and negative control (NC) groups (*p* < 0.05). However, this silencing effect diminished by day 5 (Figure 4A). Similarly, the dsAcerSmo-2 fed group also exhibited a significant interference effect compared to the negative control (NC) and sugar water (CK) groups (Figure 4B). Therefore, dsAcerSmo-1, which produced the strongest and most stable knockdown (73.3%), was selected for all subsequent transcriptomic, electrophysiological and behavioral analyses of *AcerSmo*.

**Figure 5:**
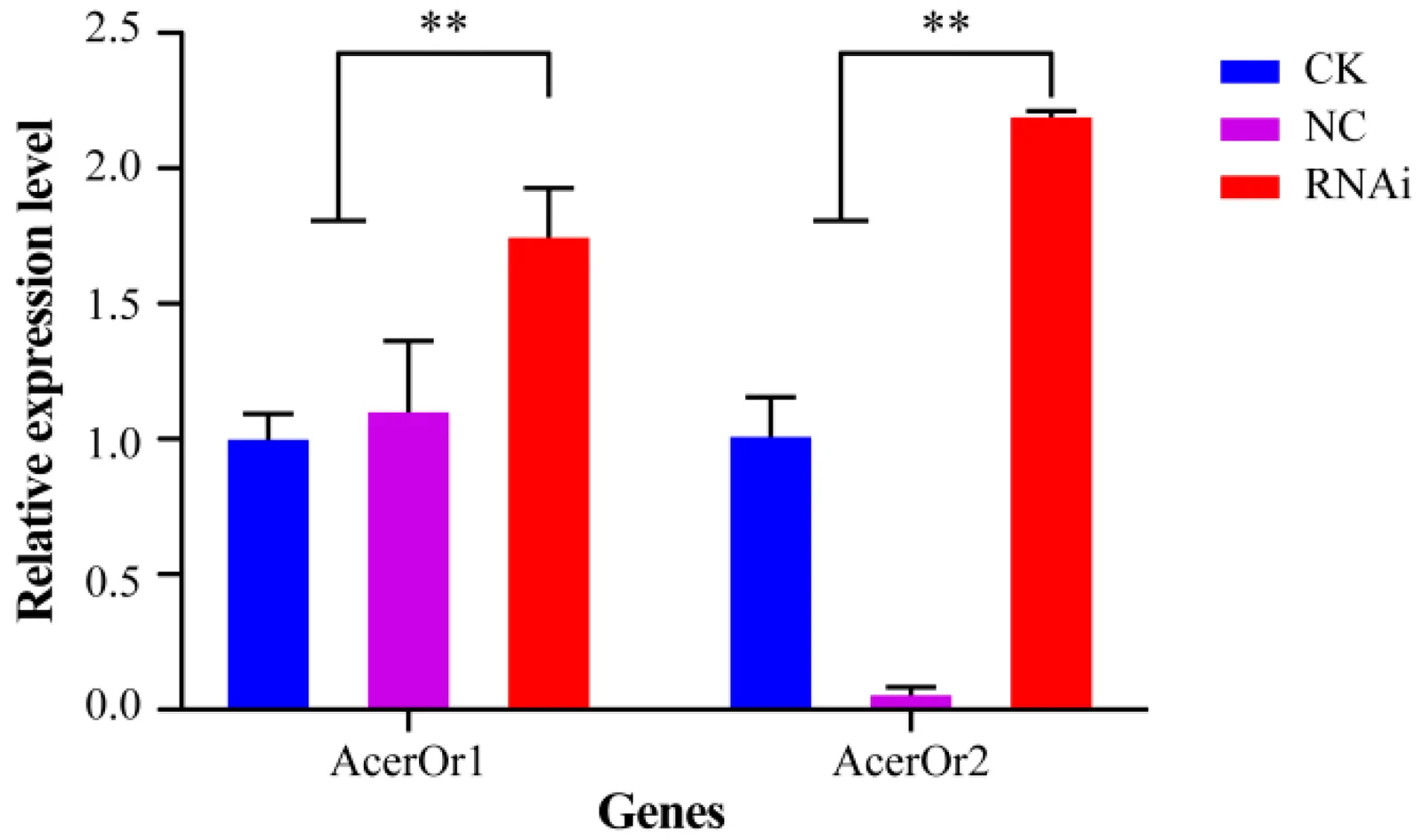
Relative expression of *AcerOr1* and *AcerOr2* genes following *AcerSmo* silencing. CK: blank control group; NC: negative control group; RNAi: gene silencing group. The y-axis represents relative gene expression level. Error bars indicate standard error of the mean, extremely significant difference (** *p* < 0.01).

RT-qPCR assays were performed on the experimental group that exhibited the most pronounced *AcerSmo* silencing effect. As depicted in Figure 5, the results demonstrated that the RNA-treated group showed significantly elevated expression levels of *AcerOr1* and *AcerOr2* compared to the control group (*p* < 0.01).

### 3.5. Evaluation of Sequencing

To investigate gene expression changes in *A. c. cerana* following the ingestion of dsRNA fragments, RNA extracted from the heads of worker bees fed with the dsAcerSmo-1 fragment for 24 h was subjected to the Illumina platform. Nine cDNA libraries were constructed: three from worker bees fed with sugar water (designated as CK1-3), three from those fed with the negative control fragment (designated as NC1-3), and three from those fed with the dsAcerSmo-1 interference fragment (designated as RNAi1-3). High-throughput sequencing yielded a total of 499,683,494 raw reads, with each sample contributing over 52,010,992 reads. Base quality was high, with Q20 content exceeding 99% and Q30 content exceeding 96% of the total bases for each sample. The average GC content was 42.67% (Table 4). Sequencing data were aligned to the *A. c. cerana* reference genome using HISAT2. For downstream analysis and annotation, only reads exhibiting perfect matches or a single mismatch with the reference genome were retained. The transcriptome data utilization rate was defined as the ratio of Mapped Reads (reads aligned to the reference genome) to Clean Reads (valid reads). The proportion of uniquely mapped reads across all samples ranged from 79.08% to 88.48%, indicating high sequencing quality and suitability for subsequent analyses (Table 5).

### 3.6. Screening of Differentially Expressed Genes (DEGs)

**Figure 6:**
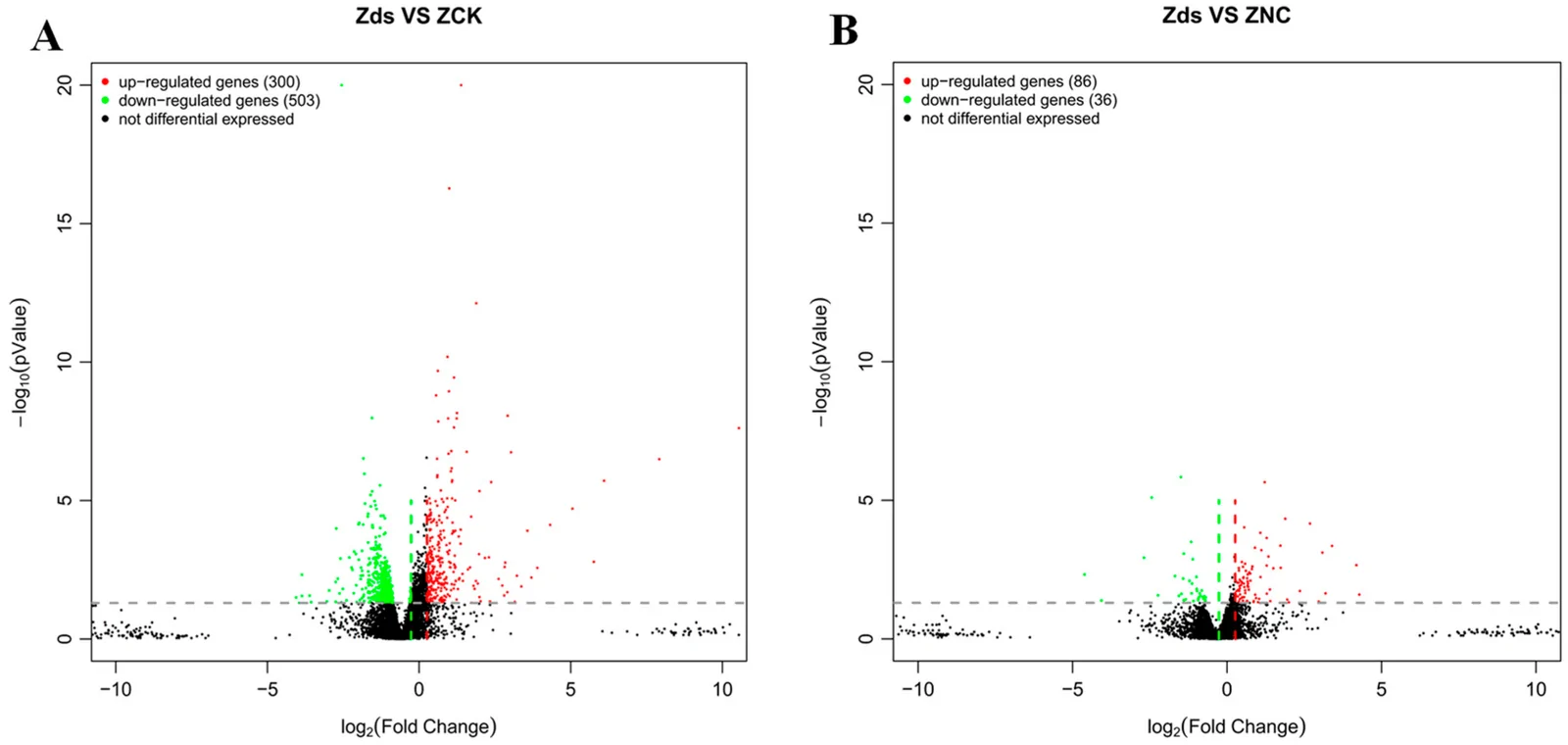
Volcano plot of expression differences between different comparison groups. Note: (**A**): Volcano plot of expression differences between CK and RNAi; (**B**): Volcano plot of expression differences between NC and RNAi.

Differential gene expression (DGE) analysis was conducted using DEGseq. This analysis identified 803 DGEs in the *A. c. cerana* RNAi-treated group compared to the CK control group. This set comprised 300 upregulated and 503 downregulated genes (Figure 6A). Furthermore, 122 DGEs were identified when comparing the RNAi treatment group to the NC control group, with 86 genes exhibiting upregulated expression and 36 genes displaying downregulated expression (Figure 6B).

### 3.7. GO Functional Annotation

**Figure 7:**
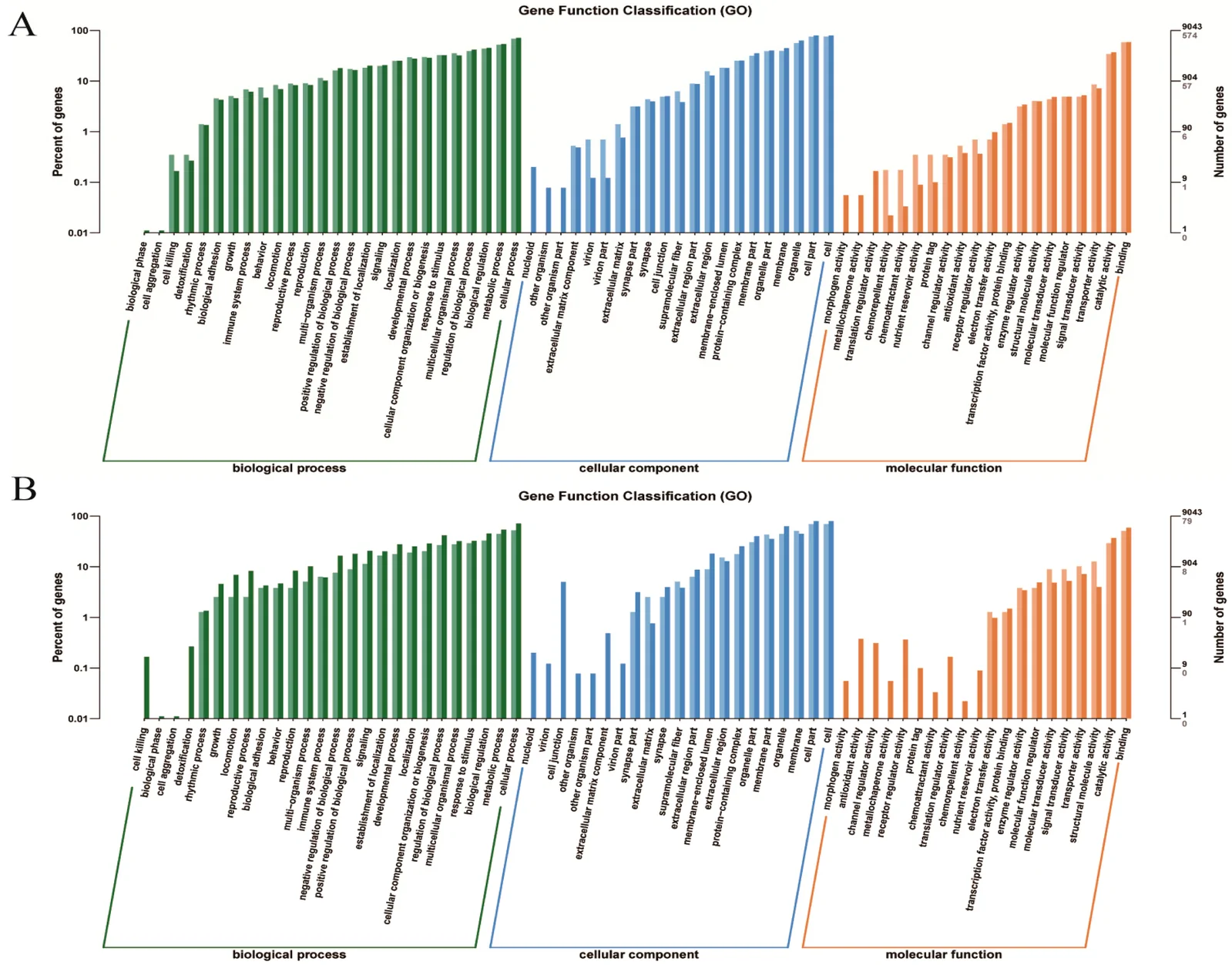
Differential Gene GO Classification. Note: (**A**): CK vs. RNAi group differential gene GO classification Chart; (**B**): NC vs. RNAi group differential gene GO classification.

Gene Ontology (GO) functional enrichment analysis was performed on the differentially expressed genes (DEGs) identified across various treatment comparisons. For the comparison between the RNAi and CK groups, a total of 5934 GO terms were found to be associated with the DEGs, comprising 4419 terms linked to biological processes, 635 to cellular components, and 880 to molecular functions. Among the top 30 significantly enriched terms, 10 were associated with biological processes and 20 with cellular components. These terms primarily focused on transcriptional regulation, regulation of protein folding and translation, phosphorylation, temperature perception, myofibril assembly, myocyte development, and proteasome regulation. Notably, prominent enriched GO terms included supramolecular fiber, supramolecular complexer, and supramolecular polymer, with the proteasome complex and the endopeptidase complex exhibiting the highest enrichment significance (Figure 7A). Subsequently, for the comparison between the RNAi and NC groups, 1472 GO terms were identified as enriched, encompassing 1024 biological processes, 209 cellular components, and 239 molecular functions. The top 30 significantly enriched terms primarily pertained to biological processes, mainly related to protein folding, transcription and translation regulation, neuromuscular development, and synapse regulation. Here, the most significantly enriched GO terms were associated with the organonitrogen compound biosynthetic process, with the cellular amide metabolic process exhibiting the highest enrichment significance (Figure 7B). Furthermore, a comparison of the top 30 enriched items from both the RNAi vs. CK and RNAi vs. NC analyses revealed five common biological process terms: protein refolding, regulation of translational initiation by eIF2 alpha phosphorylation, regulation of translation in response to stress, sarcomere organization, and myofibril assembly.

### 3.8. KEGG Functional Enrichment

**Figure 8:**
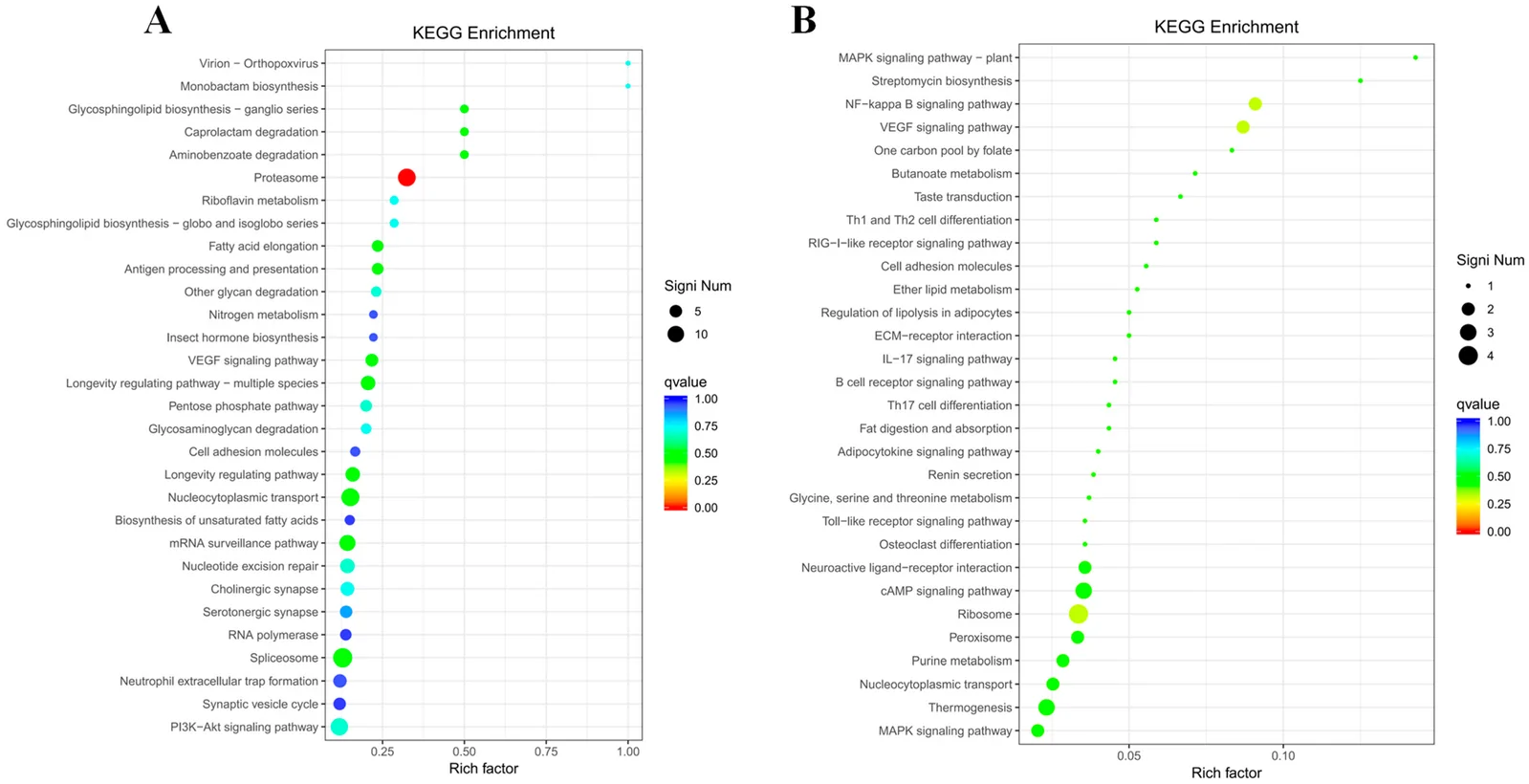
Results of KEGG enrichment of differentially expressed genes in *A. c. cerana* after RNAi. Note: (**A**): CK and RNAi groups showed different gene KEGG enrichment results; (**B**): NC and RNAi groups showed different gene KEGG enrichment results.

KEGG analysis was performed on the differentially expressed genes (DEGs) identified in each group. The KEGG analysis revealed that DEGs in the AcerSmo-RNAi treatment group, when compared to both control groups (CK and NC), were primarily enriched in four major functional categories: Genetic Information Processing, Metabolism, Environmental Information Processing, and Organism Systems (Figure 8). Among the top 30 KEGG-enriched pathways in the comparison between the CK and RNAi groups, seven were associated with Genetic Information Processing, including subcategories such as replication and repair, transcription, and protein folding, classification, and degradation. Fourteen pathways were related to Metabolism, covering lipid, amino acid, carbohydrate, nucleotide metabolism, as well as the metabolism of cofactors and vitamins. Pathways related to Environmental Information Processing included the VEGF signaling pathway, the PI3K-Akt signaling pathway, and those involving cell adhesion molecules.

The remaining 7 KEGG pathways were related to Organismal System, specifically the nervous system, immune system, and aging. Notably, the proteasome (ko03050) and nuclear transport (ko03013) pathways exhibited the highest number of enriched DEGs, with a total of 12 identified across these two pathways (Figure 8A).

For the comparison between the RNAi and NC groups, the top 30 enriched KEGG pathways revealed implications across various cellular and organismal processes (Figure 8B): Thirteen KEGG pathways were related to Metabolism, including lipid, amino acid, carbohydrate, nucleotide metabolism, and the metabolism of cofactors and vitamins. Twelve KEGG pathways are associated with Organismal Sensory processes, encompassing sensory, nervous, immune, endocrine, and digestive systems, as well as environmental adaptation, development and regeneration. Two KEGG pathways pertained to the Genetic Information, specifically transcription and translation. One KEGG pathway was related to transport and catabolism. Additionally, pathways such as NF-kappa B signaling, VEGF signaling, cAMP signaling, MAPK signaling, neuroactive ligand-receptor interaction, ECM-receptor interaction, thermogenesis, and cell adhesion molecules were categorized under Environmental Information Processing. Among these, the ribosome (ko03010) pathway contained the highest number of enriched DEGs, with 4 DEGs identified within this pathway (Figure 8B). Across both the RNAi vs. CK and RNAi vs. NC comparisons, the VEGF signaling pathway, nucleocytoplasmic transport, and cell adhesion molecules were commonly enriched among the top 30 pathways, often exhibiting with high significance.

### 3.9. Analysis of Genes Related to Olfaction

Transcriptomic sequencing analysis identified a total of 19 genes associated with olfactory behavior among the differentially expressed genes (DEGs) derived from the CK vs. RNAi and NC vs. RNAi pairwise comparisons. Among these, 8 encode olfactory receptors, 6 are involved in signal transduction, and the remaining 5 have uncharacterized functions. Differential expression analysis was performed with a false discovery rate (FDR) < 0.05 and an absolute fold change (FC) ≥ 1.2 as screening thresholds; detailed results are presented in Table 6.

Compared with the CK group, 12 out of the 19 olfactory-related genes were downregulated in the RNAi group, 10 of which showed statistically significant downregulation. Among the 7 upregulated genes, 6 exhibited significant upregulation. Similarly, relative to the NC group, 11 genes were downregulated and 7 genes were upregulated in the RNAi group, with 6 of the upregulated genes reaching statistical significance.

When comparing the two sets of pairwise differential expression results (CK vs. RNAi and NC vs. RNAi), consistent directional trends were noted for a subset of olfactory receptor genes. Specifically, Or22a-like, Or1-like, and Or13a-like showed elevated expression in the RNAi group across both comparisons, whereas Or83a-like, Or4, and Or4-like (transcript variant X1) showed reduced expression (Table 6). These expression patterns represent descriptive observations from independent pairwise analyses and have not been validated by dedicated cross-group statistical testing.

GO enrichment analysis revealed two shared categories between the CK vs. RNAi and NC vs. RNAi groups: olfactory behavior (a biological process) and olfactory receptor activity (a molecular function). Specifically, for the CK vs. RNAi group, 6 DEGs were enriched in olfactory behavior, comprising 4 upregulated and 2 downregulated genes. Conversely, in the NC vs. RNAi group, all 3 enriched DEGs associated with olfactory behavior were upregulated.

Regarding olfactory receptor activity, the CK vs. RNAi comparison showed enrichment in 12 DEGs (4 upregulated, 8 downregulated). In contrast, for the NC vs. RNAi group, all 5 DEGs enriched in olfactory receptor activity were upregulated. Additionally, olfactory learning (a biological process) was enriched with 3 DEGs in the CK and RNAi comparison, consisting of 2 upregulated and 1 downregulated gene. KEGG pathway analysis revealed one downregulated DEG enriched in olfactory transduction within the CK vs. RNAi comparison. Notably, no olfactory-related pathways were enriched in the NC and RNAi comparison, possibly due to lower expression levels of olfactory-related genes.

### 3.10. DEGs Were Verified by qRT-PCR

The RNA-Seq results for olfactory-related genes in *A. c. cerana* were validated using RT-qPCR. Both methods confirmed the upregulation of the uncharacterized gene LOC107996980, guanine nucleotide-binding protein subunit gamma-e, and synapsin, whereas Or83a-like, Or4, and Or4-like transcript variant X1 showed downregulation (Table 7). These concordant results partially validated the RNA-seq results and supported the overall reliability of the transcriptomic analysis.

### 3.11. EAG Response to Compounds After RNAi Silencing

**Figure 9:**
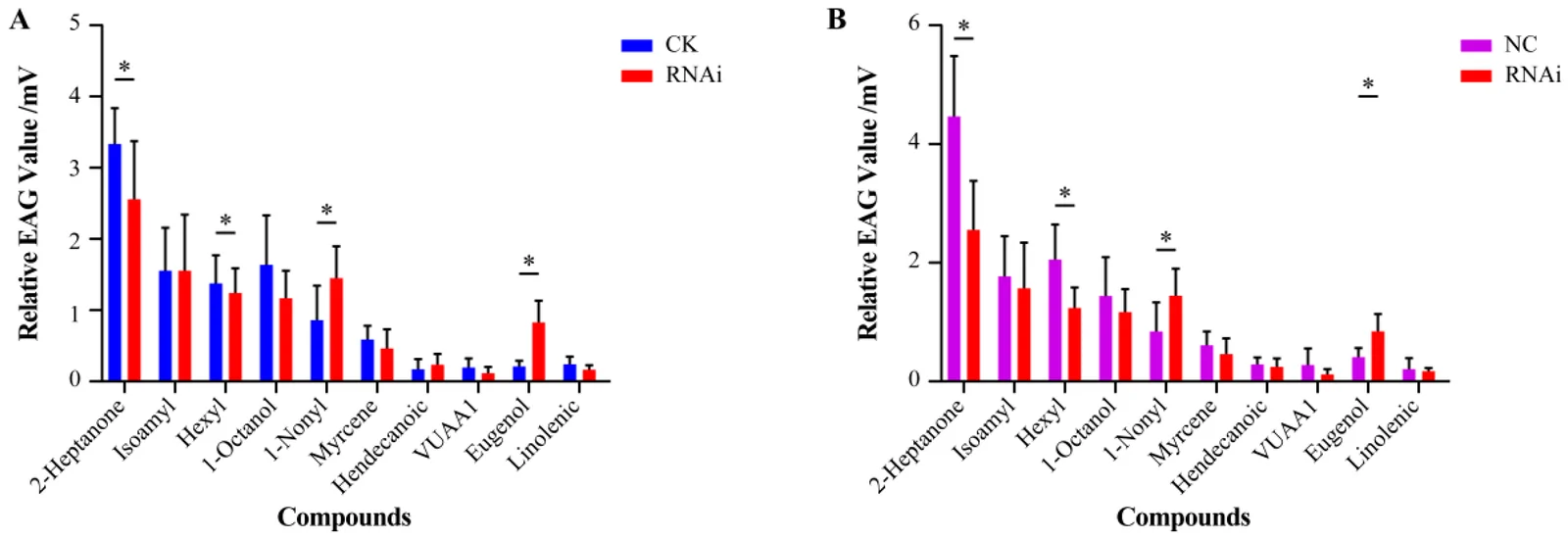
EAG responses of *A. c. cerana* to different odorants before and after RNAi treatment. (**A**) Relative EAG values of bees in CK and RNAi groups. (**B**) Relative EAG values of bees in NC and RNAi groups. CK, blank control; NC, negative dsRNA control; RNAi, dsAcerSmo injection group. The y-axis represents relative EAG amplitude (mV). Error bars indicate standard error of the mean. * indicates significant difference (*p* < 0.05).

After RNA interference treatment, electroantennogram (EAG) experiments were conducted (Figure 9). Compared with the CK (sugar water control) group and the NC (negative control) group, the EAG responses of *A. c. cerana* antennae to 2-heptanone and n-hexyl acetate were significantly lower in the RNAi-treated group (*p* < 0.05), whereas the EAG responses to 1-nonanol and eugenol were significantly higher (*p* < 0.05).

### 3.12. Behavioral Tests After RNAi Silencing

**Figure 10:**
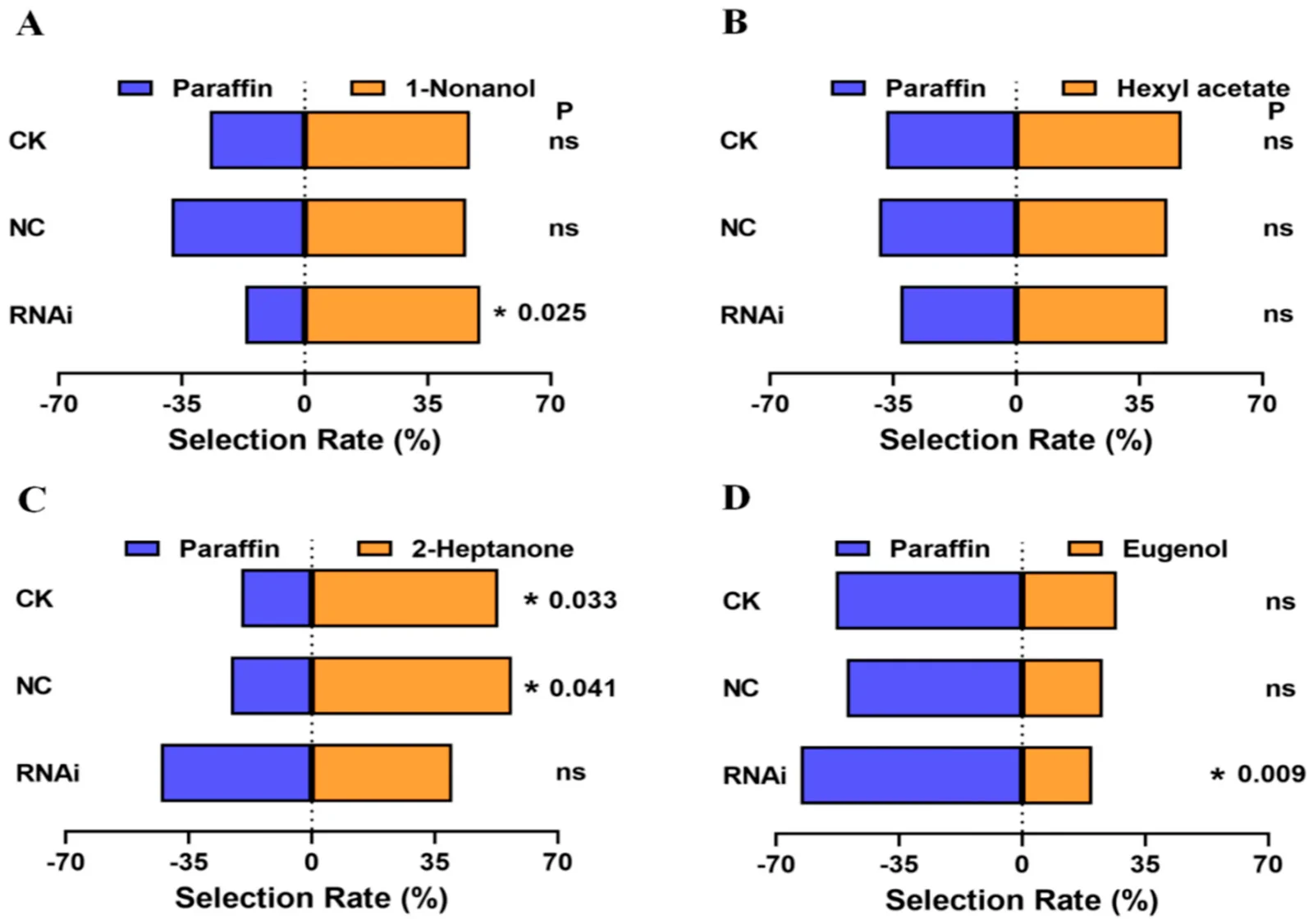
Selectivity of *A. c. cerana* for different odorants before and after RNAi. (**A**) Choice rate between paraffin oil and 1-Nonanol; (**B**) Choice rate between paraffin oil and Hexyl acetate; (**C**) Choice rate between paraffin oil and 2-Heptanone; (**D**) Choice rate between paraffin oil and Eugenol. CK, blank control group; NC, negative dsRNA control group; RNAi, dsAcerSmo injection group. The x-axis indicates selection rate (%). * represents significant difference (*p* < 0.05), ns indicates no significant difference.

Based on our research group’s preliminary experimental results, 2-heptanone, hexyl acetate, 1-nonanol, and eugenol were selected for Y-tube olfactometer testing. The results showed that before *AcerSmo* gene interference, there was no significant difference in the selection rates of *A. c. cerana* between 1-nonanol and paraffin (*p* > 0.05); however, after interference, the selection rate for 1-nonanol significantly increased (*p* < 0.05) (Figure 10A). Before *AcerSmo* gene interference, *A. c. cerana* exhibited significant selectivity for 2-heptanone (*p* < 0.05); whereas after interference, there was no significant difference in selection rates between paraffin and 2-heptanone (*p* > 0.05) (Figure 10C). Compared with the control groups (CK and NC), interference with the *AcerSmo* gene enhanced the avoidance of *A. c. cerana* toward 2-heptanone. Before *AcerSmo* gene interference, there was no significant difference in the selection rates of *A. c. cerana* between eugenol and paraffin (*p* > 0.05); after interference, the selection rate for eugenol significantly decreased (*p* < 0.05) (Figure 10D). Before and after interference, no significant differences were observed in the selection rates of *A. c. cerana* between hexyl acetate and paraffin (*p* > 0.05) (Figure 10B).

## 4. Discussion

Smo is essential for transmitting Hedgehog (Hh) signals within the cell. Under normal conditions, Smo is inhibited when bound to Patched at the cell membrane. However, when Hh binds to Patched, it triggers the endocytosis of the Hh-Patched complex. This event releases Smo, enabling it to transmit the Hh intracellularly. The apparent discrepancy between *AcerSmo* transcript localization and protein distribution should be interpreted cautiously. Although this pattern is compatible with cell-type-specific post-transcriptional regulation, alternative explanations such as differential protein stability, intracellular trafficking, delayed translation, or technical limitations cannot be excluded. Future studies using spatial transcriptomics, ribosome profiling or live-cell imaging will be required to determine whether intercellular RNA transport occurs.

In *Mus musculus*, the Hh signaling pathway has been observed to modulate odor responses by regulating the accumulation of odorant receptors (ORs) on cilia [24]. Similarly, insect antennae are equipped with a multitude of sensory receptors that facilitate the nuanced and precise detection of external stimuli. Notably, the transduction of olfactory signals in insects is contingent upon the binding of odorant molecules to olfactory receptors located within these antennal sensors [25]. The antennal sensilla of Hymenoptera, for instance, comprise a diverse array of structures, including sensilla trichodea (without pores, sensilla trichodea with a tip pore), sensilla coeloconica, sensilla placodea, and sensilla chaetica [26,27].

In honey bees, sensilla trichodea, sensilla placodea, and sensilla coeloconica are the predominant forms of antennal sensilla [28]. Sensilla placodea are ubiquitous on insect antennae, housing the majority of olfactory sensory neurons (OSNs), and their role in olfaction is well-established through various studies [29,30,31]. The surface layer of sensilla trichodea is punctuated by numerous small pores, believed to facilitate the reception of diverse specific odor molecules from the environment. Consequently, sensilla trichodea are intimately associated with olfactory recognition [30]. In situ hybridization results showed that *AcerSmo* mRNA is widely distributed within the cells of each subsegment of the antennal flagellum of *A. c. cerana*, with signals specifically enriched in the neuronal layer beneath the base of both trichoid and placoid sensilla, suggesting that *AcerSmo* is transcriptionally expressed in the sensory neurons associated with these sensillum types. This distribution pattern indicates that *AcerSmo* may play a role in olfactory recognition. Furthermore, the Smo protein has been shown to regulate olfactory recognition in *Drosophila* by controlling odorant receptor trafficking through its subcellular localization within cilia [9]. Based on this evidence, we speculate that Smo may similarly regulate odorant receptors and thereby influence olfactory perception in honey bees. Immunohistochemical analysis further revealed the localization pattern of the *AcerSmo* protein. *AcerSmo* protein was expressed in the scape, pedicel, and flagellum, with higher protein levels detected in the proximal subsegments of the flagellum, consistent with the abundant sensilla distribution in these regions [14]. At the sensillum level, *AcerSmo* protein was localized to non-neuronal supporting cells (likely accessory cells associated with olfactory sensory neurons) in trichoid and plaoid sensilla, whereas it was expressed within the coeloconic sensilla. This result is markedly different from the pattern of mRNA expression detected by in situ hybridization, which showed localization in sensory neurons. In-depth analysis suggests that this discrepancy arises from a unique transcription–translation cellular segregation mechanism inF trichoid and placoid sensilla: the transcription of the *AcerSmo* gene occurs exclusively in olfactory sensory neurons, but after transcription, the resulting mRNA is not directly translated within the neuronal cell bodies. Instead, it is transferred to adjacent non-neuronal supporting cells through intercellular molecular transport pathways, where protein synthesis and accumulation take place, ultimately leading to spatial separation of mRNA and protein expression across different cell types [32,33]. This heterogeneous expression of transcription and translation further supports the existence of distinct functional regulatory pathways for *AcerSmo* in different sensillum types: in trichoid and placoid sensilla, *AcerSmo* employs a “neuronal transcription–supporting cell translation” mode, indirectly regulating olfactory signal transduction in olfactory sensory neurons via supporting cells. In contrast, in coeloconic sensilla, both transcription and translation of *AcerSmo* occur within cells located inside the sensillum, with no intercellular molecular transport, thereby maintaining consistency between mRNA and protein localization. This allows *AcerSmo* to directly participate in chemosensory signal transduction within the sensillum, ultimately achieving complex regulatory roles for *AcerSmo* in multi-type chemoperception in honey bees.

This method is gentler for bees, more feasible to implement, and causes almost no harm to them [34]. In this study, *AcerSmo* was successfully silenced in *A. c. cerana*, with the most pronounced silencing effect observed one day post-feeding, and no significant mortality after five days of feeding.

To investigate the effects of different treatments on gene expression, transcriptome sequencing was performed on head (including antennae) samples of *A. c. cerana* fed with the interference fragment (dsAcerSmo-1), a negative control (NC) fragment, and a sugar water control (CK). It is noteworthy that substantially more differentially expressed genes were identified in the CK versus RNAi comparison than in the NC versus RNAi comparison. This difference suggests that, in addition to the silencing effect of *AcerSmo*, dsRNA administration itself or the feeding procedure may elicit a limited transcriptional response in honey bees. Nevertheless, the much smaller number of DEGs between the NC and RNAi groups indicates that the majority of the observed transcriptional changes are more likely attributable to *AcerSmo* knockdown rather than nonspecific effects of dsRNA treatment. GO enrichment analysis of the resulting differentially expressed genes indicated their primary enrichment in biological processes such as transcription and translation, material transport, neuromuscular development, and stress response. KEGG enrichment analysis demonstrated significant enrichment in pathways related to VEGF signaling, nucleocytoplasmic transport, and cell adhesion. Additionally, notable involvement was observed in various metabolic processes, including those for lipids, amino acids, carbohydrates, and nucleotides. The Smo protein, categorized within the G protein-coupled receptor (GPCR) family, is predicted to have a seven-transmembrane architecture. Previous studies indicate its predominant localization to the endoplasmic reticulum and nucleus, consistent with its role in signal transduction. The significant functional enrichment of DEGs in processes such as transcription, translation, signal transduction, material transportation, nucleocytoplasmic transport, and cell adhesion molecules suggests that the *AcerSmo* protein is involved in transmembrane signal transduction and material transport. In both insects and mammals, the Smo protein is crucial for tissue development and regeneration processes [35]. The significant enrichment of DEGs in neuromuscular development, organism development and senescence, and the VEGF signaling pathway further suggests a crucial role for *AcerSmo* in tissue development and regeneration in *A. c. cerana*. GO analysis results indicated that interference with *AcerSmo* leads to the enrichment of olfactory-related DEGs associated with olfactory receptor activity and behavior. This suggests that the *AcerSmo* protein plays a role in influencing olfactory behavior through the modulation of receptor activity. Through the observed alterations in gene expression levels, this study revealed that the *AcerSmo* protein has the potential to impact olfactory behavior by modulating the function of olfactory receptors. Despite this finding, the precise mechanism of action remains elusive. Given the intricate regulatory network governing olfactory behavior, it is plausible that the *AcerSmo* protein may also be involved in additional regulatory pathways. In addition to olfactory receptor-related genes, *AcerSmo* knockdown altered genes involved in proteasome activity, nucleocytoplasmic transport, protein processing and cellular metabolism. These pathways may influence odor perception indirectly by regulating receptor turnover, intracellular trafficking of signaling molecules, and protein homeostasis within olfactory sensory neurons, thereby contributing to the electrophysiological and behavioral phenotypes observed after *AcerSmo* silencing in AcerSmo*A. c. cerana*.

Studies have shown that sterols can directly activate Smo and disruption of sterol biosynthesis may impact Hh signal transduction [36]. Several steroid-binding sites are present on the Smoothened protein, with the cysteine-rich domain (CRD) identified as the primary binding site. It is hypothesized that Smo is activated through cholesterol binding to the CRD, suggesting cholesterol as a potential endogenous agonist for Smo [36]. The significant enrichment of lipid metabolism observed in the KEGG analysis may stem from the disruption of *AcerSmo*. Furthermore, studies have shown that Smo gene knockout downregulates glutaminase, the rate-limiting enzyme in glutamine catabolism [37]. In this study, RNA interference (RNAi) was implemented by feeding double-stranded RNA (dsRNA). The DEGs show enrichment in metabolic pathways such as amino acid and carbohydrate metabolism. ORs play a crucial role in olfactory signal transduction. Among the odorant receptor genes identified in this study, Or1-like exhibited increased expression post-RNAi treatment. This suggests that the *AcerSmo* protein exerts inhibitory effects on *AcerOr1* and related genes in *A. c. cerana*. Ultimately, olfactory behavior is regulated by multiple ORs.

To verify the reliability of the RNA-seq data, several DEGs were selected for RT-qPCR validation. The RT-qPCR results showed expression trends that were generally consistent with those obtained by RNA-seq, supporting the overall reliability of the transcriptomic analysis. However, noticeable differences in fold-change magnitude were observed for several genes, such as synapsin and LOC107996980. These discrepancies may reflect differences in detection sensitivity, amplification efficiency, normalization strategies, and dynamic range between RNA-seq and RT-qPCR, which are commonly observed when comparing the two techniques. Therefore, the RT-qPCR results should be regarded as a partial validation of the transcriptomic data rather than complete confirmation of all quantitative expression changes.

*A. c. cerana* possesses functional odorant receptors, *AcerOr1* and *AcerOr2*. *AcerOr1* is associated with the detection of floral scents, while *AcerOr2* is essential for olfactory signal transduction [17,18]. The expression of *AcerSmo* exerts a suppressive effect on the olfactory receptor genes *AcerOr1* and *AcerOr2*, thereby playing a restrictive role. RT-qPCR data revealed that the mRNA levels of both *AcerOr1* and *AcerOr2* were significantly upregulated following *AcerSmo* silencing.

After interfering with the *AcerSmo* gene in *A. c. cerana*, electroantennogram (EAG) experiments showed significantly reduced responses to 2-heptanone and hexyl acetate, suggesting that *AcerSmo* positively regulates the olfactory receptors responsible for binding these compounds, promoting their interaction with corresponding ligands. Following *AcerSmo* interference, this regulatory effect is weakened, leading to reduced receptor–ligand binding and consequently diminished EAG responses. Studies have shown that at physiological concentrations, 2-heptanone typically induces avoidance behavior in honeybees [14]. In this study, interference with *AcerSmo* resulted in enhanced avoidance behavior of *A. c. cerana* toward 2-heptanone. Therefore, we hypothesize that the protein encoded by *AcerSmo* may be involved in negatively regulating the sensitivity of honeybees to 2-heptanone. Under normal conditions, this gene exerts an inhibitory role in the olfactory pathway to finely regulate the intensity of avoidance behavior and prevent overreaction. Interference with the gene removes this inhibition, leading to abnormally enhanced avoidance behavior. Studies have shown that 2-heptanone is an alarm pheromone secreted by the mandibular gland of honeybees [38,39]. At abnormally high concentrations, it triggers defensive responses, whereas at physiological concentrations, it primarily induces repellent behavior [14,37]. Vallet found that the content of 2-heptanone in the mandibular gland of a single worker of *A. c. cerana* varies with age and division of labor, ranging from approximately 0.1 to 7 microliters (μL). The concentration of 2-heptanone used in this experiment was 500 μg/μL, which falls within a relatively high concentration range. Although this concentration is still within the physiological range reported in some literature, the direct stimulation using a single droplet in the experiment effectively simulated a localized high-concentration exposure, which may lead honeybees to perceive it as a threat signal rather than a conventional alarm pheromone. Therefore, we speculate that, in the absence of *AcerSmo* gene interference, the significantly higher approach behavioral response of *A. c. cerana* toward 2-heptanone compared to paraffin (control), one possible explanation is that the behavioral responses to 2-heptanone may be associated with its multiple ecological functions reported in previous studies. However, the present study did not directly investigate the neural mechanisms underlying this response. Therefore, this interpretation should be regarded as a working hypothesis, and further experimental evidence is required to verify the underlying mechanism [36]. Among the tested odorants, after *AcerSmo* interference, the relative EAG responses of *A. c. cerana* to 1-nonanol and eugenol significantly increased. Furthermore, behavioral assays showed that the selection rate of *A. c. cerana* toward 1-nonanol was enhanced, while the avoidance rate toward eugenol was enhanced. Combined with qPCR results showing that *AcerSmo* interference significantly upregulated the expression levels of *AcerOr1* and *AcerOr2*, we hypothesize that the recognition of 1-nonanol in *A. c. cerana* may be regulated by *AcerOr1* and *AcerOr2*. The significantly reduced selectivity of *A. c. cerana* toward eugenol following RNA interference suggests that *AcerSmo* may negatively affect the ability of honeybees to recognize this odorant. In insect olfactory behavior research, inconsistency between EAG responses and behavioral outcomes is not uncommon [40]. Insect choice behavior toward volatiles can be influenced by multiple physiological and environmental factors, which may explain the observed discrepancies. *AcerSmo* may differentially modulate odor-specific signaling pathways, resulting in both increased and decreased EAG responses depending on the odorant tested. Because the underlying molecular mechanisms remain unclear, further studies are required to determine how *AcerSmo* regulates the sensitivity of different olfactory pathways.

The majority of olfactory stimuli encountered in nature are complex mixtures composed of numerous unique scents. The identities and quantities of these individual components are pivotal for elucidating odor-mediated behavior [41]. While we assessed the electrophysiological and behavioral responses of *A. c. cerana* to several individual scents, both prior to and following RNAi treatment, future research should aim to confirm any changes in honey bee olfactory behavior by systematically combining these monomers at varying ratios. Because electrophysiological recordings were conducted using a single odor concentration, concentration-dependent effects could not be evaluated. Future studies should establish dose–response curves to distinguish changes in odor sensitivity from possible saturation effects.

Through the observed alterations in gene expression levels, this study revealed that the *AcerSmo* protein has the potential to impact olfactory behavior by modulating the function of olfactory receptors. Despite this finding, the precise mechanism of action remains elusive. Given the intricate regulatory network governing olfactory behavior, it is plausible that the *AcerSmo* protein may also be involved in additional regulatory pathways.

## 5. Conclusions

Our findings indicate that *AcerSmo* is involved in the olfactory recognition process of *A. c. cerana*. In situ hybridization revealed that *AcerSmo* mRNA is expressed in the sensory neurons beneath trichoid and placoid sensilla, while immunohistochemistry showed that *AcerSmo* protein is localized to non-neuronal supporting cells in these sensillum types, suggesting a unique transcription–translation segregation mechanism. Functional assays demonstrated that *AcerSmo* suppresses the expression of the olfactory receptor genes *AcerOr1* and *AcerOr2*, and influences the detection of specific odorants, including eugenol, 1-nonanol, and 2-heptanone. Transcriptome sequencing further identified 19 olfaction-related genes affected by *AcerSmo* silencing, among which nine are OR-related and five are involved in signal transduction, with Or1-like showing upregulated expression following RNAi treatment. GO and KEGG analyses revealed that differentially expressed genes are enriched in multiple biological processes, including transcription and translation, signal transduction, substance transport, neuromuscular development, sensory and neural system function, as well as lipid, amino acid, carbohydrate, and nucleotide metabolism. Collectively, these results suggest that *AcerSmo* not only participates in olfactory signal modulation by regulating olfactory receptor expression but may also influence a wide range of physiological processes that indirectly support olfactory function in *A. c. cerana*.

## Figures and Tables

**Table 1:** Primers used in this study.

Gene	Primer Sequence (5′–3′)	Amplicon Size (bp)	Purpose
dsAcerSmo-1	F: GCCAGAUGAACAUAAACAATT		RNAi
	R: UUGUUUAUGUUCAUCUGGCTT		
dsAcerSmo-2	F: GAAACUGAAGAACCUAUUATT		
	R: UAAUAGGUUCUUCAGUUUCTT		
*NC*	F: UUCUCCGAACGUGUCACGUTT		
	R: ACGUGACACGUUCGGAGAATT		
AcerSmo	F: ACCGGAAGAAGAAGCAAAGAT	3699	qPCR
	R: CTCTGCTCCGTCTATCACTTTC		
*Arp*1	F:TGCTGCACTCGTAGTTGACAATGG	1172	
	R: ACCCTGGTGGCGTGGTCTTC		
*AcerOr1*	F: ATCTTCTTCGCATTCCACG	1507	
	R: ATGAAAGTGATTGCCGCTC		
*AcerOr2*	F: GTGTTGTTCTGCTCCTGGCT	1.763	
	R: GGAAGGTGGTCGTGAAGTCG		

**Table 2:** Standard compounds used in this study.

Compounds	CAS Number	Purity	Origin
Undecanoic acid	112-37-8	>99.0% (GC)	Macklin, Shanghai, China
Eugenol	97-53-0	>99.5% (GC)	Macklin, Shanghai, China
2-Heptanone	110-43-0	≥99.8% (HPLC)	Aladdin, Shanghai, China
Isoamyl acetate	123-92-2	>99.5% (GC)	Aladdin, Shanghai, China
Linolenic acid	463-40-1	>98% (GC)	Aladdin, Shanghai, China
Hexyl acetate	142-92-7	≥99.5% (HPLC)	Macklin, Shanghai, China
Myrcene	123-35-3	≥99.0 (HPLC)	Macklin, Shanghai, China
n-Octanol	111-87-5	≥99.5% (HPLC)	Macklin, Shanghai, China
1-Nonanol	143-08-8	>99.5% (GC)	Macklin, Shanghai, China
VUAA1	525582-84-7	≥98% (HPLC)	Macklin, Shanghai, China

**Table 3:** Detection results of the purified antibody.

Number	Dilution	Purified Anti-AcerSmo Antibody
		Optical Density at 450 nm
1	500	4.000
2	1500	4.000
3	4500	3.648
4	13,500	3.648
5	40,500	2.369
6	121,500	1.144
7	364,500	0.583
8	1,093,500	0.346
9	3,280,500	0.293
10	9,841,500	0.283
11	29,524,500	0.271
12	Blank control	0.258
	Titer	>364,500

**Table 4:** The RNA-seq data assessment statistics.

Sample	Raw Data	Total Bases Count (bp)	Q20 = (%)	Q30 (%)	GC (%)
CK1	60,862,122	9,129,318,300	99.05	96.27	41.71
CK2	57,535,394	8,630,309,100	99.13	96.61	44.16
CK3	55,138,636	8,270,795,400	99.13	96.59	41.77
NC1	58,661,682	8,799,252,300	99.18	96.77	44.17
NC2	52,628,758	7,894,313,700	99.04	96.21	41.67
NC3	52,010,992	7,801,648,800	99.00	96.09	42.06
RNAi1	54,092,974	8,113,946,100	99.17	96.73	42.14
RNAi2	54,636,944	8,195,541,600	99.12	96.54	42.31
RNAi3	54,115,992	8,117,398,800	99.03	96.20	43.56

**Table 5:** Sequencing data and reference genome sequence comparison.

Sample	Clean Reads	Total Mapped	Uniquely Mapped (%)	Multiple Mapped (%)
CK1	57,416,622	93.14%	88.48	4.67%
CK2	54,544,686	93.40%	87.54	5.86%
CK3	52,416,470	92.38%	87.41	4.97%
NC1	56,091,166	92.56%	87.14	5.42%
NC2	49,535,072	86.78%	82.73	4.06%
NC3	48,902,364	92.92%	88.33	4.59%
RNAi1	51,070,140	85.63%	81.07	4.57%
RNAi2	52,129,332	84.01%	79.09	4.93%
RNAi3	51,159,876	93.04%	87.99	5.05%

**Table 6:** Expression of significantly differentially expressed genes in different comparison groups.

Gene ID	Gene Name	RNAi vs. CK	RNAi vs. NC
LOC114576785	odorant receptor 83a-like	Down (sign.)	Down
LOC107996720	odorant receptor 4	Down (sign.)	Down
LOC107996744	odorant receptor 4-like	Down (sign.)	Down
LOC107999608	uncharacterized	Down (sign.)	Down
LOC114576851	uncharacterized	Down (sign.)	Down
LOC108003836	synapsin	Down (sign.)	Down
LOC107992650	uncharacterized	Down (sign.)	Down
LOC114577388	odorant receptor 22a-like	Up (sign.)	Up (sign.)
LOC114577389	uncharacterized	Down	Up (sign.)
LOC107996749	odorant receptor 4-like, transcript variant X1	Down (sign.)	Down
LOC107993405	odorant receptor Or1-like, transcript variant X1	Up	Up (sign.)
LOC107996980	uncharacterized	Up (sign.)	Up
LOC107996975	uncharacterized	Up (sign.)	Up (sign.)
LOC107996106	filamin-A, transcript variant X1	Up (sign.)	Down
LOC107993163	odorant receptor 22c-like	Down (sign.)	——
LOC107992669	odorant receptor 13a-like	Up (sign.)	Up (sign.)
LOC108004356	cAMP-specific 3′,5′-cyclic phosphodiesterase-like	Up (sign.)	Down
LOC108004440	guanine nucleotide-binding protein subunit gamma-e	Down (sign.)	Down
LOC107995099	integrin beta-nu-like, transcript variant X1	Down	Up (sign.)

Note: Up indicates upregulation of the gene in the RNAi group; Down indicates downregulation of the gene in the RNAi group; —— indicates no difference.

**Table 7:** Verification of differentially expressed genes in *A. c. cerana* using qRT-PCR.

Gene	Fold by RNA-Seq	Fold by qRT-PCR
uncharacterized LOC107996980	1.20	1.11
guanine nucleotide-binding protein subunit gamma-e	−1.25	−0.36
synapsin	−0.10	−0.82
odorant receptor 83a-like	−2.96	−2.22
odorant receptor 4	−1.11	−1.15
odorant receptor 4-like transcript variant X1	−1.17	−0.13

## Data Availability

The RNA-seq datasets generated and analyzed during the current study have been deposited in the NCBI Gene Expression Omnibus (GEO) database under accession number GSE324143. The datasets are currently under embargo. RNA-seq raw data are also available in the NCBI Sequence Read Archive (SRA) under BioProject accession number PRJNA1196307. The GenBank accession number of the *AcerSmo* gene is OP616035. GenBank accession numbers for *AcerArp1*, *AcerOr1*, and *AcerOr2* are HM640276.1, JN792580, and JN792581, respectively. All other data supporting the findings of this study are available from the corresponding author upon reasonable request.

## Abbreviations

Smo	Smoothened
EAG	Electroantennography
Hh	Hedgehog
Ors	odorant receptors
Orco	odor coreceptor
OSNs	olfactory sensory neurons
SRA	Short Read Archive
TPM	Transcripts Per Million
DEGs	differentially expressed genes
GO	Gene Ontology
KEGG	Kyoto Encyclopedia of Genes and Genomes
